# Implementing the Global Plan to Stop TB, 2011–2015 – Optimizing Allocations and the Global Fund’s Contribution: A Scenario Projections Study

**DOI:** 10.1371/journal.pone.0038816

**Published:** 2012-06-18

**Authors:** Eline L. Korenromp, Philippe Glaziou, Christopher Fitzpatrick, Katherine Floyd, Mehran Hosseini, Mario Raviglione, Rifat Atun, Brian Williams

**Affiliations:** 1 Global Fund to Fight AIDS, Tuberculosis and Malaria, Geneva, Switzerland; 2 Department of Public Health, Erasmus Medical Centre, University Medical Center Rotterdam, Rotterdam, The Netherlands; 3 World Health Organization, Geneva, Switzerland; 4 Imperial College London, London, United Kingdom; 5 South African Centre for Epidemiological Modelling and Analysis (SACEMA), Stellenbosch, South Africa; Johns Hopkins Bloomberg School of Public Health, United States of America

## Abstract

**Background:**

The Global Plan to Stop TB estimates funding required in low- and middle-income countries to achieve TB control targets set by the Stop TB Partnership within the context of the Millennium Development Goals. We estimate the contribution and impact of Global Fund investments under various scenarios of allocations across interventions and regions.

**Methodology/Principal Findings:**

Using Global Plan assumptions on expected cases and mortality, we estimate treatment costs and mortality impact for diagnosis and treatment for drug-sensitive and multidrug-resistant TB (MDR-TB), including antiretroviral treatment (ART) during DOTS for HIV-co-infected patients, for four country groups, overall and for the Global Fund investments. In 2015, China and India account for 24% of funding need, Eastern Europe and Central Asia (EECA) for 33%, sub-Saharan Africa (SSA) for 20%, and other low- and middle-income countries for 24%. Scale-up of MDR-TB treatment, especially in EECA, drives an increasing global TB funding need – an essential investment to contain the mortality burden associated with MDR-TB and future disease costs. Funding needs rise fastest in SSA, reflecting increasing coverage need of improved TB/HIV management, which saves most lives per dollar spent in the short term. The Global Fund is expected to finance 8–12% of Global Plan implementation costs annually. Lives saved through Global Fund TB support within the available funding envelope could increase 37% if allocations shifted from current regional demand patterns to a prioritized scale-up of improved TB/HIV treatment and secondly DOTS, both mainly in Africa − with EECA region, which has disproportionately high per-patient costs, funded from alternative resources.

**Conclusions/Significance:**

These findings, alongside country funding gaps, domestic funding and implementation capacity and equity considerations, should inform strategies and policies for international donors, national governments and disease control programs to implement a more optimal investment approach focusing on highest-impact populations and interventions.

## Introduction

The Global Plan to Stop TB for 2011 to 2015 (Global Plan), developed by the Stop TB Partnership with major inputs from the World Health Organization (WHO), sets out the level of tuberculosis (TB) interventions and funding that National TB Control Programs (NTPs) will need to reach Millennium Development Goal (MDG) 6 TB targets and the related targets set by the Stop TB Partnership for 2015 (see next section). The Plan’s cost projections are based on estimates of TB disease and deaths, intervention targets and service implementation costs for low- and middle-income countries (LMIC) [1].

Between 2003 and 2011, NTPs that had received funding from the Global Fund had provided treatment to 8.6 million people with sputum smear-positive TB [2]. In 2011, the Global Fund provided 76% of the external financing for TB and multi-drug-resistant TB (MDR-TB) control in LMIC and 11% of the total funding for TB in these countries [3]. Between its launch in 2002 and the end of 2011, the Global Fund invested US$2.3 billion in TB grants in 116 countries, and disbursed in 2010 US$512 million in TB grants [4]. In 2010, the Global Fund estimated donor pledges and projected income of US$11.7 billion for investment in HIV, TB and malaria over 2011 to 2013 [5].

The Global Fund fosters a demand-driven model of co-funding NTPs. Demand is moderated by the technical support provided to countries by international health agencies when developing proposals to request funds, followed by an independent technical expert review of these proposals on soundness and local appropriateness of interventions and programs. By 2010, this grant making model had resulted in an investment pattern among countries in line with their overall share in burden, for each of the three diseases [6].

Based on control priorities identified by technical agencies such as the WHO, the Global Fund regularly updates its guidance to countries on effective interventions it will finance. The Global Fund Strategy 2012–2016 which commits to scaling-up TB interventions according to the Global Plan [7] emphasises efficient allocation of available financial resources to achieve maximum health impact as measured by infections prevented, deaths averted and lives saved. In this context, we explore allocation scenarios in which available Global Fund financing could contribute to implementing the Global Plan through 2015. We first estimate total NTP costs and lives saved, separately for (i) diagnosis and DOTS treatment of fully drug-susceptible TB cases (hereafter DOTS), (ii) diagnosis and treatment of multi-drug-resistant TB (hereafter MDR-TB), and (iii) antiretroviral treatment (ART) during DOTS treatment for HIV-coinfected patients (hereafter TB/HIV care), if Global Plan targets were fully met. To estimate the Global Fund’s contribution, a base-case scenario allocates TB grants among countries and regions following the demand-based pattern observed over 2007 to 2009 [8]. We assume that implementing NTPs would allocate grant funding between DOTS, MDR-TB treatment and TB/HIV care, in proportion to their needs for these services as determined in the Global Plan. Against this base-case, we then evaluate different scenarios, in which a changed distribution of grants among countries and services either maximizes lives saved, or prioritizes the regions with the largest burden of TB/HIV or MDR-TB.

### The Global Plan to Stop TB, 2011–2015: Targets and Financing Requirements

The overall goal of the Global Plan to Stop TB is to achieve the MDG and Stop TB partnership targets set for 2015 [1]:

TB in the *Millennium Development Goals* (set for 2015):

Goal 6: Combat HIV/AIDS, malaria and other diseases

- Target 6c: Halt and begin to reverse the incidence of malaria and other major diseases;
- Indicator 6.9: Incidence, prevalence and death rates associated with TB;
- Indicator 6.10: Proportion of TB cases detected and cured under DOTS.

*Stop TB Partnership* targets (set for 2015 and 2050)

- By 2015: Reduce prevalence and death rates by 50%, compared with their levels in 1990;
- By 2050: Eliminate TB as a public health problem, defined as a global incidence of active TB of less than one case per 1 million population per year.

Specific Global Plan targets for 2015, according to the major components of the plan, include:

- DOTS: 6.9 million cases diagnosed, notified and treated according to the DOTS approach, in 2015 alone (compared to a baseline of 5.8 million in 2009 alone), with 90% of drug-susceptible cases cured (compared to an actual treatment success rate of 86% in high-burden countries at end-2009).
- TB/HIV: Expand the enrolment of HIV-coinfected TB patients on ART, such that by 2015 all HIV-positive TB patients – estimated at almost 1 million people in 2009– are enrolled on ART. This target compares to 140,000 actual ART enrolments per year as (an estimated 37% of need) of mid-2010.
- MDR-TB: Diagnosis and treatment of 270,000 MDR-TB patients in 2015, i.e. 61% of estimated total MDR-TB cases in 2008, with 100% of confirmed cases treated according to international guidelines and an increase in the treatment success rate of confirmed MDR-TB from the 2009 baseline of 60% to ≥75% by 2015 [1]. In comparison, in 2008 [1] and 2009 [9] 5% and 12% of MDR-TB cases had been detected, respectively.

These targets have been based on a situation analysis of actual progress in service delivery that NTPs made over 2006–2010. Absolute numbers of treatments corresponding to these targets, with a gradual scale-up from 2011 to 2015, are based on WHO country estimates of the current burden of drug-susceptible, MDR-TB and TB/HIV cases, projected forward to 2015 in a log-linear model fitted to the 2005–2009 observed trend [9], [10].

The corresponding funding requirements are an overall US$ 47 billion over 2011 to 2015, including almost US$ 37 million for implementation and almost US$ 10 billion for research and development. Specific funding requirements include:

- DOTS: US$ 22.6 billion
- MDR-TB: US$ 7.1 billion
- TB/HIV: US$ 2.8 billion.

In 2011, more than 40 countries reported to have used the WHO’s TB planning and budgeting tool [44] in planning their national strategies in line with recommendations and targets of the Global Plan, and in 2011, all the 27 high-MDR-TB countries produced new national MDR-TB plans in line with the Global Plan.

## Methods

### Interventions and Countries Considered

We use Global Plan projections of anticipated numbers of TB cases that will be detected in DOTS programs led by NTPs in the period 2011 to 2015, and WHO estimates of TB cases in 2010 as a baseline [1]. We focus on low- and middle-income countries, as high-income countries are ineligible for Global Fund financing.

We used WHO country trend estimates, based on notification and treatment outcome data reported annually by NTPs, to project expected TB cases in the different patient categories of DOTS, MDR-TB and TB/HIV care, and feasible numbers of these detected and treated in each country in the Global Plan [9], [10]. Projections assume that Global Plan targets and funding requirements are fully met, that all TB cases detected by NTPs are first treated for drug-susceptible TB, and that a sub-set of these subsequently get tested and treated for MDR-TB or receive ART if co-infected with HIV. When we present estimates of numbers treated and lives saved, the three patient categories are mutually exclusive so the number of drug-susceptible TB cases does not include MDR-TB or patients with active TB and HIV co-infection. Cost projections for DOTS, however, include DOTS treatments for all cases including those with MDR-TB and TB/HIV, as all patients would have received DOTS first. The costs labelled MDR and ART are then the additional costs of treating MDR-TB or TB/HIV, over and above the costs incurred for the DOTS treatment.

To explore different allocation scenarios, we divided countries into four groups with distinct epidemiological and TB burden characteristics, namely:

1. China and India, which together account for about a third of all incident TB cases worldwide [9];
2. Eastern Europe and Central Asia (EECA), where the prevalence of MDR-TB among TB cases is highest;
3. sub-Saharan Africa (SSA), where prevalence and impact of TB/HIV is highest;
4. all other low- and middle-income countries (Table S1).

### Per-patient Costs in NTP Budgets

Funding needs are based on country-specific needs and targets as determined in the Global Plan, which also estimated the per-person cost of DOTS, MDR-TB, and treatment and care for HIV-co-infected TB patients, including ART for the duration of DOTS treatment, in each group of countries (Table 1). Cost assumptions (Table 2) were based on expenditure data and national program budget projections reported by NTPs to WHO [9]; for MDR-TB treatment the cost estimation furthermore included costing studies in selected countries [11], [12], [13].

**Table 1:** Assumed per-patient cost (US $) of projected TB interventions by country group.

Countries	Number of countries	DOTS	MDR treatment	6 months of ART for TB/HIV patient
China and India	2	503	4,315	271
Eastern Europe and Central Asia	16	5,582	9,299	273
Sub-Saharan Africa	46	503	4,315	236
Other low- and middle-income countries	85	503	4,315	250

Notes: Stated amounts reflect unit costs in US$ as of 2010, of (i) diagnosing and treating one TB patient under DOTS [1] and (ii) the additional cost incurred if the patient has multi-drug resistant (MDR) TB as estimated in the WHO/Stop TB partnership Global Plan to Stop TB 2011–2015 [1], and (iii) the additional cost incurred if the patient is HIV-positive and receives antiretroviral therapy (ART) for the duration of a 6-month DOTS course [47]. Costs are inflated at 3% per annum. Regional cost estimates were based on country cost estimates, weighted by each country’s notified incident cases.

**Table 2:** Cost components borne by NTPs, included in the Global Plan to Stop TB, 2011–2015.

DOTS	Laboratory diagnosis: sputum smears, including scale-up of fluorescent light-emitting diode microscopy to replace conventional light microscopy, and X-rays
	First-line drugs
	Health workers and NTP staff
	Programme management
	Practical Approach to Lung Health
	Private Public Mix
	Community-based Care
	Advocacy Communications and Social Mobilization
	Operational research and surveys
MDR-TB	Second- and third-line drugs
	Hospitalization including infection control
	DOT visits
	Sputum smears, cultures, drug susceptibility testing with scale-up in the use of liquid culture media to replace solid media
	Training, programme and data management
	Provision of food parcels
TB/HIV	Antiretroviral treatment for the six months’ duration of DOTS treatment, the period that TB and HIV treatment overlap. Initiation of ART during DOTS treatment is a highly cost-effective, WHO-recommended intervention to reduce early mortality [45], [46]

Notes: In addition to DOTS, management of MDR-TB and TB/HIV, the Global Plan includes estimates of costs for co-trimoxazole preventive therapy (CPT) during DOTS, nutritional support, HIV serological testing and counselling for HIV-coinfected patients, and isoniazid-based preventive therapy (IPT) to prevent HIV-positive people with latent *Mycobacterium tuberculosis* infection from developing active TB disease [1]. Our projections do not include these added costs, which are relatively small for CPT (e.g. less than $10 per patient-year in Uganda [48]), difficult to express per TB patient for IPT, which concerns HIV-infected patients *without* active TB, and not necessarily borne by NTPs for nutritional support and for HIV testing and counselling. Globally, uptake of IPT remains low, in spite of efforts by normative and financing agencies to increase its implementation [49]. One factor contributing to this slow uptake is the absence of sensitive and specific tests distinguishing between active disease and latent TB [49]; other factors warrant further exploration by the major normative and financing agencies for TB control.

### Lives Saved

Lives saved were estimated using case fatality rates with and without treatment for each category of patients. We chose a no-treatment counterfactual for all three treatment categories, to validly compare these interventions for their full potential health benefit. The global average case fatality rates were derived from published studies and systematic reviews identified through a PubMed search, up to December 2011 [1], [9], [10], [14], [15], [16], and treatment outcome data reported by NTPs to WHO. Key search terms included “Tuberculosis”, “Mortality” and “Case fatality”. Relevant additional studies were obtained by screening reference lists from relevant articles, and from the WHO’s TB library, including publications in English and French as described in more detail in [1], [9], [10], [11], [12], [14], [15], [16].

Against a counterfactual of no treatment, every DOTS treatment of a drug-susceptible HIV-uninfected case is assumed to avert 0.33 deaths [15], and every treatment with ART of an HIV-coinfected patient 0.50 deaths [14], [16]. For MDR-TB, there are no published empirical data on case fatality without treatment. We estimated the average lives saved per MDR-TB treatment as being 0.30, i.e. 90% of the lives saved per DOTS treatment, based on the typical lower cure rates for MDR-TB than for DOTS, as reported by NTPs to WHO [1], [9].

Uncertainty ranges in lives saved were estimated to reflect the wide variation among countries and populations in case fatality rates, which is apparent from published research studies, and from treatment outcomes reported by NTPs [9]. Model-based ranges are ±40% to ±60% for the fatality rate within each case category [10], [17], [18], [19]; we applied an error of 50% to each fatality rate.

### NTP Budgets and Funding Sources

Table 3 shows the sources of current funding for TB control in each region in 2010. NTP budgets by source were based on data reported to WHO in 2009 [9], [20]. At aggregate level, domestic funding accounted for 76% to 97% of total TB funding available, including that from the Global Fund and other international contributions. Sub-Saharan Africa (77% domestic overall, but 48% when South Africa is excluded), and China and India (76%) are at the lower end of this range, whereas EECA covers 97% of TB funding from domestic resources. Similarly, when domestic TB expenditures are expressed as a proportion of Gross Domestic Product (GDP), sub-Saharan Africa, and China and India spend well below other regions, notably below EECA.

**Table 3:** Sources of funding for TB control, according to NTP preliminary 2010 budgets.

Amounts in millions of US$	China and India	Eastern Europe & Central Asia	Sub-Saharan Africa	Other low and middle-income countries	All low and middle-income countries
General health services	38	416	371	625	**1,450**
Government	230	1,540	273	327	**2,370**
Global Fund	63	67	124	133	**387**
Other grants	20	2	58	38	**117**
**Total available**	**351**	**2,025**	**826**	**1,123**	**4,324**
Domestic/Total	76%	97%	78%	85%	**88%**
Domestic/GDP	0.004%	0.094%	0.067%	0.012%	**0.022%**
Need 2015	1,912	2,562	1,564	1,850	**7,888**
Regional share of Global Fund TB disbursements	16%	17%	32%	34%	**100%**

Notes: Preliminary NTP budgets for 2010 were reported to WHO by 107 of the 149 Global Plan countries, which together accounted for 98% of the global burden of TB in 2009 [9]. According to these figures, $3.8 billion was available from domestic sources in 2010. This domestic contribution included approximately $1.5 billion spent on general inpatient and outpatient health services, outside of NTP budgets, which were estimated based on costs and frequencies of hospital admissions and outpatient visits to health facilities by TB patients [9], [20].

Government: national governments including loans; Grants: external donors excluding the Global Fund; Total available = general health services + Government + Global Fund + Other grants. Need: total TB control need, as defined in the 2010 Global Plan to Stop TB. Domestic = General health services + Government; GDP = gross domestic product (purchasing power parity); Regional share of Global Fund = proportion of worldwide Global Fund TB disbursements going to each region, average 2007 to 2009.

Projections of TB program expenditures funded by the Global Fund were based on 2010 donor pledges and projected income of US$11.7 billion for the replenishment period 2011 to 2013. Using the regional distribution of Global Fund disbursements from 2007 to 2009 [6], we assumed that 16% of financing would be allocated to TB over the next five years, with the rest allocated to HIV/AIDS (49%), malaria (34%) and health systems strengthening (captured within the three disease areas). Projections assume a one-year time lag between disbursements and program expenditures.

### Allocation Scenarios

From 2007 to 2009, 32% of Global Fund TB disbursements were in sub-Saharan Africa, 16% in India and China, 17% in EECA, and 34% in other LMIC (Table 4). For the base-case projections (**Scenario A**), we assumed that these regional allocation patterns are maintained from 2011 to 2015. Base-case projections furthermore assumed that, within each region, Global Fund investments would be allocated to DOTS, MDR-TB treatment and ART in proportion to their respective funding needs according to the Global Plan. **Scenario B** then considers the effect of re-allocating TB investments among regions and interventions to maximize lives saved by Global Fund financing. For comparison, a **Scenario C** explores what would happen if the Global Fund’s policy were to first exclusively finance DOTS with concurrent ART for HIV-coinfected TB patients, and next MDR-TB treatment, before contributing to DOTS for HIV-negative TB patients – as under the Global Plan, treatment of HIV-coinfected TB patients and MDR-TB both require a more rapid funding increase than does DOTS [21].

**Table 4:** Percentage distribution of funding need for implementing DOTS, MDR-TB treatment and ART during DOTS, over regions in 2015.

		DOTS	MDR	ART	All treatments	
China and India		18.0	5.5	0.7	**24.2**	
Eastern Europe andCentral Asia		20.3	12.1	0.1	**32.5**	
Sub-Saharan Africa		13.8	1.8	4.2	**19.8**	
Other low- and middle-income countries	18.9		4.0	0.6		**23.5**
**All low- and middle-** **income countries**		**71.0**	**23.4**	**5.6**	**100.0**	

Notes: The projected total funding need for the three services in 2015 is US$7.9 billion according to the Global Plan to Stop TB 2011–2015 [1]. *DOTS* is the cost of first-line DOTS for all TB cases including those with MDR-TB and/or coinfected with HIV. *MDR* is the *additional* cost for treating those with MDR-TB and *ART* the *additional* cost for treating those that are HIV-positive with ART for six months during DOTS.

## Results

### Service Coverage and Funding Needs

**Figure 1:**
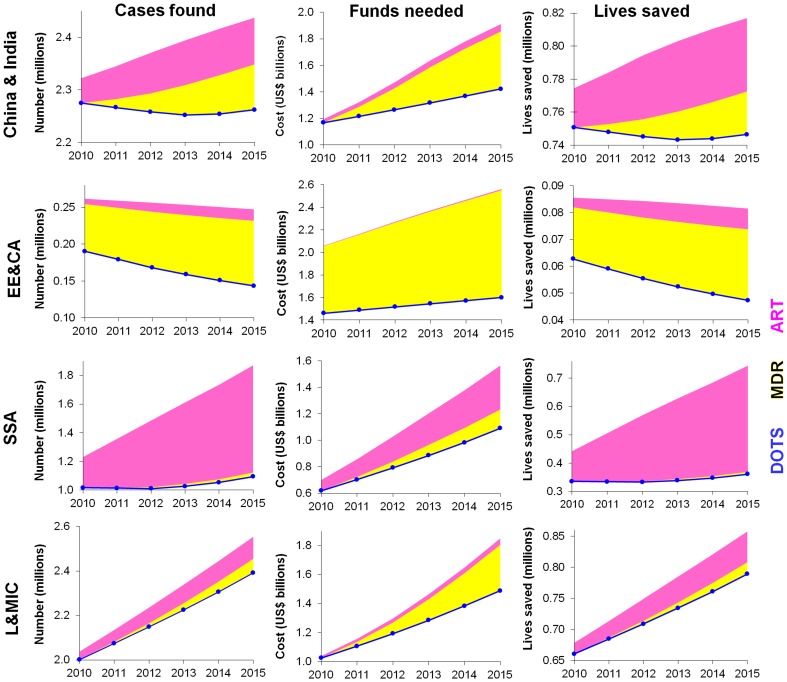
Cases of drug-susceptible TB, MDR-TB and HIV-related TB that will be found according to the Global Plan to Stop TB (left); corresponding funding need (centre); and corresponding lives saved (right). Notes to Figure 1: Global Plan forecasts based on date reported by NTPs to WHO up to 2009 [1], [9]. Rows top to bottom: C&I: China and India; EE&CA: EECA; sub-Saharan Africa (SSA); L&MIC: Low- and middle-income countries not included in the other three regions. The cost of DOTS for drug-susceptible TB, MDR-TB and TB/HIV patients is included in ‘DOTS’ (blue circles & lines); yellow and pink bars cover the additional cost of providing MDR treatment or ART during DOTS treatment. *Note that vertical axes do not start from zero.*

Overall Global Plan targets for LMIC in 2015 [1] are 6.9 million DOTS treatments for drug-susceptible TB, including 1.0 million people living with HIV, and 0.27 million MDR-TB treatments (Box) [1]. To meet Global Plan targets across LMIC, the total funding needed for the three interventions increases from $5.0 billion in 2010 to $7.9 billion in 2015 (Figure 1). In comparison, Global Plan cost estimates for overall control implementation total just over $6 billion in 2011 and rise to $8.5 billion in 2015, reflecting additional cost of co-trimoxazole preventive therapy, nutritional support for HIV-coinfected patients, HIV testing and counselling, and isoniazid-based preventive therapy for HIV-positive people with latent *Mycobacterium (M.) tuberculosis* infection [1] (Table 2).

### Regional Needs

In **China and India** (Figure 1: top), DOTS treatments fall slowly, TB/HIV cases increase slowly (reflecting a stable, low prevalence of HIV), but there is a large increase in patients treated for MDR-TB. Funding needed increases from US$1.2 billion in 2010 to US$1.9 billion in 2015, mainly reflecting the cost of treating MDR-TB in increasing numbers of people.

In **EECA** (Figure 1: second row) total treatments fall slowly, but the decline in drug-susceptible TB will be balanced by increasing MDR-TB. Since MDR-TB is more costly to treat, overall funding need will increase by 25% between 2010 and 2015. By 2015, 62% of total funding will be allocated to drug-susceptible TB, 37% to MDR-TB and less than 1% to TB/HIV.

**Sub-Saharan Africa** (Figure 1: third row) accounts for the largest number of people with TB/HIV among regions. In 2015, 40% of TB patients will be HIV-positive, but only about 2% will have MDR-TB. The rapid increase in DOTS need reflects mainly HIV-positive TB patients needing DOTS together with ART; numbers of HIV-negative TB patients remain fairly constant.

In the **other** **low- and middle-income countries** (Figure 1: fourth row), the greatest cost remains for treating drug-susceptible TB (US$1.4 billion in 2015). Funding needed for MDR-TB increases from near 0 in 2010 to US$317 million in 2015.

Across the four regions, in 2015 EECA will need the most treatment funding and sub-Saharan Africa the least (Figure 1, middle column and Table 4). In all regions the greatest need remains for DOTS, the cost of which increases slightly over the years, as per-patient costs rise [1]. However, in EECA 37% of funding is needed for MDR-TB with a minimal need for TB/HIV, whereas in sub-Saharan Africa 21% is needed for ART in HIV-coinfected patients.

### Lives Saved

Over 2011−15, scaling up treatment will increase the annual lives saved from 2.1 to 2.5 million (uncertainty ranges: 1.0 to 3.1 million, and 1.3 to 3.8 million, respectively), reflecting mainly increasing treatments among HIV-coinfected patients (Figure 1). MDR-TB treatment accounts for 4% of lives saved, corresponding to its small share of treatments.

Among regions, about 800,000 lives (uncertainty range: 400,000 to 1,200,000) will be saved in each of China and India, sub-Saharan Africa and other low- and middle-income countries. Only about one-tenth as many lives will be saved in EECA, with fewer patients than China & India or sub-Saharan Africa. Nevertheless, this region needs most funding because of its large proportion of MDR-TB treatments, and the high per-patient cost for both drug-susceptible TB and MDR-TB (Table 1). The largest annual increase in lives saved is in sub-Saharan Africa, where expected intervention coverage increases most rapidly. In EECA, in contrast, annual lives saved decrease slightly from 2010 to 2015, reflecting the decline in DOTS treatment need following the ongoing fall in case notifications.

### Global Fund Contribution to TB Funding

**Figure 2:**
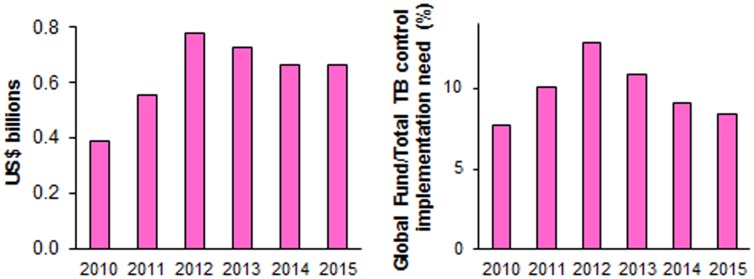
Global Fund contribution to TB control, low- and middle-income countries. (A): Expected Global Fund TB expenditures; (B): Corresponding proportional share in the total funding need for DOTS, MDR-TB and TB/HIV treatment. Note to Figure 2: Projections based on October 2010 donor pledges for 2011−2013.The projected decline after 2012 is larger for the Global Fund’s proportional contribution than for its absolute TB expenditures, as global TB funding needs continue to rise through 2015.

Based on 2010 donor pledges and projected income, and assuming the continuation of recent TB/HIV/malaria funding allocation patterns, Global Fund TB investments will increase from US$387 million in 2010 to $779 million in 2012, and then fall to US$662 million in 2015 (Figure 2A). The peak in 2012 and subsequent decline in annual expenditures reflects commitment patterns over 2011−2013, of which the majority is used to renew existing grants, with relatively smaller amounts available for new programs [8].

In 2010, the Global Fund’s total TB expenditures of $387 million covered 7.7% of DOTS, MDR, and TB/HIV funding need in LMIC (Figure 2B). This contribution would increase to 12.8% in 2012, then decline to 8.4% by 2015.

### Global Fund Investment Scenarios Modelled

**Figure 3:**
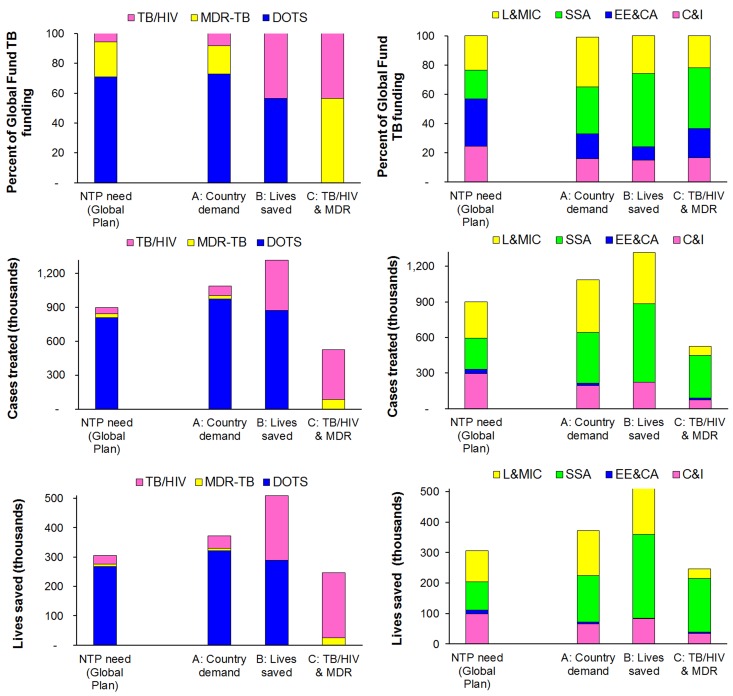
Global Fund TB allocations (top), corresponding cases treated (middle) and lives saved (bottom), across services (left) and regions (right), for three scenarios in 2015. Notes to Figure 3: **Scenario A** assumes that regional allocations remain in the distribution of 2007–9 approved funding, with allocations among services following regional distributions of need according to the Global Plan to Stop TB. **Scenario B** maximizes mortality impact per dollar spent. **Scenario C** allocates money to DOTS+ART for TB/HIV patients and to MDR-TB treatment only. For comparison, left-most bars show results if grant distributions would exactly match total national funding needs as projected in the Global Plan. C&I: China and India; EE&CA: EECA; L&MIC: other low and middle-income countries; SSA: sub-Saharan Africa.

In base-case **Scenario A**, regional TB investments are aligned with the regional distribution in total need (Figure 3a). Global Fund financing would save on average 265,000 lives per year, reaching 373,000 in 2015 (uncertainty range 186,000−559,000; Figure 3c). Lives saved from DOTS, MDR-TB and TB/HIV treatment (Figure 3c) are roughly proportional to treatment numbers in these respective categories (Figure 3b). Only TB/HIV treatments account for a larger proportion of lives saved compared to their share of patients, because of the higher case fatality of untreated TB/HIV co-infection compared to drug-susceptible TB or MDR-TB without HIV.

**Scenario B** maximizes mortality impact from the funding available. Of the three services, DOTS with concurrent ART for TB/HIV patients saves most lives per dollar, within the 6-months TB treatment duration considered as time horizon, in each region; therefore this scenario first allocates enough grant money to fully finance DOTS with concurrent ART for all HIV-coinfected patients (Figure 3a). In 2015 alone, DOTS with concurrent ART for TB/HIV patients worldwide would cost US$742 million, absorbing 73% of the projected Global Fund resources for TB in that year. The remaining 27% of available TB funding is then allocated to treat HIV-uninfected, drug-susceptible TB in sub-Saharan Africa, China and India, and other LMIC. If DOTS allocations are distributed among these three regions in proportion to TB grants received over 2007–9, this will cover 19% of DOTS need in sub-Saharan Africa, 8% in China and India, and 17% in the other LMIC. This scenario would substantially increase allocations to SSA countries (Figure 3a) and decrease allocations to EECA, supported for just ART for their small number of HIV-coinfected patients without Global Fund support for MDR-TB or DOTS. Scenario B increases total lives saved in 2015 by 37% to 510,000 (255,000−765,000; Figure 3c).

In **Scenario C**, as in Scenario B, allocations covers DOTS with ART for all HIV-coinfected TB patients, but remaining funding is then allocated to MDR-TB in proportion to 2007–9 regional grant patterns (Figure 3). Compared to the base-case, scenario C increases allocations to SSA by 29%, slightly increases allocations to EECA, and decreases allocations to other countries (Figure 3a). A similar number of lives (222,000; range 111,000−332,000) are saved among HIV-coinfected TB patients as in **Scenario B**, but since all remaining funds are invested on costly MDR-TB treatment mainly in EECA (Figure 3b), total lives saved are one-third lower than in the base-case (Figure 3c).

## Discussion

Our study is the first to synthesize worldwide data and estimates of TB treatments, cost and funding from different sources, and mortality impact expected over 2011 to 2015 in countries worldwide, intoscenarios to optimize TB funding allocations between regions and services. The projections show how the cost of global TB control is driven by MDR-TB treatment need, mainly in EECA region, and by the future overall efficiency and effectiveness of TB treatment in this region.

During the past decade of DOTS expansion, the burden of drug-susceptible TB cases has stabilized, with a slow decline in *per capita* rates, and the total number of annual new drug sensitive TB cases globally are falling since 2006. Especially in India and China annual numbers of drug-susceptible cases are projected to decline between 2011 and 2015 [9], following considerable improvement in DOTS programmes over the past decade [22] (Figure 1). In contrast, MDR-TB treatments are projected to increase in all regions, as the Global Plan targets a significant improvement in the proportion of MDR-TB cases detected and treated, from just 12% in 2009 [9] to 61% by 2015 [1]. The high per-patient cost and comparatively lower cure rates for MDR-TB (as compared with DOTS) provides a low immediate rate of return, especially in EECA region. However, the health and economic consequences of poorly controlled MDR-TB are grave with global spread and massive future treatment costs that risk undermining global TB control, and investing in MDR-TB diagnosis and care is highly cost-effective in the longer term [12], [13], [23].

Increasing investment is needed to scale up treatment of TB/HIV co-infection, especially in Africa. HIV testing and counselling at start of TB treatment is an important entry point to find HIV-positive people in need of ART or pre-ART care. Among TB/HIV-coinfected patients, investment in ART has a disproportionally high rate of return as it greatly reduces mortality during DOTS [24]. When considering the cost of ART for just the six months of DOTS, ART is not disproportionally expensive, and even in sub-Saharan Africa corresponds to only 20% of total TB funding need – and in practice, not all HIV-coinfected TB patients will start ART right away at DOTS enrolment. For a given amount of funding, the global lives saved are therefore strongly influenced by the balance in investment allocations between TB/HIV management mainly in Africa, with relatively high return for low cost, and MDR-TB management mainly in EECA, with lowest immediate direct return for highest cost. For countries where both TB/HIV coinfection and MDR-TB are highly prevalent, such as South Africa, the presented analysis implies that an important priority should remain to prevent MDR from occurring and spreading in the first place, by good-quality DOTS at high coverage, including among HIV-coinfected patients who require concurrent ART for the DOTS treatment to be successful.

### Global Fund Investments in TB Control

If low- and middle-income countries maintain their share of financing dedicated to TB control in line with GDP growth, domestic funding will amount to US$6.5 billion annually by 2015, based on GDP projections from the International Monetary Fund (IMF) [25]. Under a more conservative scenario with domestic TB funding increasing proportionally with GDP *per capita*, US$5.2 billion domestic funding would be available for TB control in 2015. More conservative yet, if keeping pace just with inflation, domestic funding would total US$4.5 billion in 2015. In all cases, domestic funding will substantially fall short of the Global Plan need (US$8.5 billion in 2015 alone for control implementation, excluding research and development) – underscoring the importance of continued co-financing by the Global Fund and other donors.

Model projections show that the Global Fund could potentially increase the health impact of its TB investments, if allocations prioritized Africa and TB/HIV (Scenario B). Of all regions, Africa has the highest immediate return on TB investment, because of its high prevalence of TB/HIV co-infection, and relatively low per-patient costs. An increased donor focus on sub-Saharan Africa is also reasonable given the rapid rise in TB funding need from 2010 to 2015, of 82% (1.82-fold, Figure 1) much higher than the rate at which most African countries will be able to expand domestic contributions.

Alternatively, the prospect of controlling MDR-TB might improve with preferential allocations to MDR-TB treatment (Scenario C). This policy would, however, reduce lives saved compared to the base-case, because the high per-patient cost for MDR-TB treatment compared to DOTS, and the high per-patient costs in eastern Europe and Central Asia, the region with most MDR-TB, reduce the total number of treatments affordable for the given amount of total funding. Affordability of TB control globally will critically depend on the ability in EECA to improve NTP effectiveness and efficiency (for both DOTS and MDR-TB), by transitioning from the current hospital-centered service delivery [9], [26], [27] to WHO-recommended cost-effective implementation systems based on good-quality DOTS delivered through primary outpatient facilities, thus improving cure rates and preventing the emergence of MDR-TB [28].

Along with EECA countries, also India, China and other rapidly advancing economies like Brazil and South Africa may be expected to assume progressively greater domestic co-financing of TB control, as their national incomes grow and TB burden decreases [22]. The IMF forecasts that China and India will increase their *per capita* GDP by 72% and 50%, respectively, from 2010 to 2015. Considering their projected 60% increase in TB funding need over this period, the expected economic growth would enable maintaining or increasing domestic contributions relative to GDP – currently below those in other regions (Table 3). Nevertheless, to complement local government funding TB control may require continued donor co-funding for special service areas, including for civil societies that are only just organizing themselves, public-private mix activities, and to strengthen community systems enhancing take-up of new services by the poorest, most vulnerable people [29], [30].

### Limitations

Several limitations must be considered in interpreting findings. First, estimates and projections of TB case incidence and mortality are uncertain, given limitations in data especially for the highest-burden countries. The WHO and Global Plan estimates nevertheless represent the most up-to-date, coherent set of estimates, based on best available data from research studies and NTPs, expert consensus and consultations with 90 countries [9], [10].

Second, there is uncertainty about future Global Fund expenditures, as well as on how this funding will increase the level and quality of TB services. Projections assumed that Global Fund expenditures will peak in 2012 [8], but this may shift as donor contributions to the Global Fund vary over the period 2011−15. Based on TB disbursements actually made in 2010 and 2011 and foreseen (as of April 2012), the 2010 Replenishment-based projection was accurate for 2010 and 2011, but it may have somewhat overestimated TB expenditures expected in 2012 and 2013 due to delays in donor payments relative to 2010 pledges. Also, from 2012 onwards, each supported country must make a minimum domestic government co-funding contribution relative to the Global Fund’s budget, of a proportion increasing with national income and with the years of each grant [31]. In this context of transitioning to self-sustainability, the projections’ assumption that the Global Fund bears the full cost for a given number of treatments will in the future become less and less relevant. Especially in upper-middle income countries, Global Fund grants will increasingly provide only a share of costs (e.g. for drugs, laboratory or selected other program components), with the remainder financed from domestic and other donor resources. The Global Fund’s 2011 updated eligibility, prioritization and counterpart financing policy further exclude from new grant agreements upper-middle income countries that are part of the G-20 [31] – so that notably China is expected to transition out from Global Fund TB support from mid-2013, and Russia from end-2013. In general, the increased counterpart financing requirements on especially upper-middle income countries should result in a gradual shift of portfolio allocations toward lower-income countries. A strategic re-allocation of Global Fund TB grants from higher-capacity EECA to poorer and needier countries would, however, require additional policies, since EECA already fund 97% of NTP budget themselves.

Projections furthermore assumed ART during DOTS to be financed through the Global Fund’s TB funding envelope. In practice this service is largely financed through HIV grants, so the total Global Fund financial contribution to TB control is actually greater than presented here.

The 2012 call for proposals called ‘Transitional Funding Mechanism’ [32], to fund continuation of selected essential prevention, treatment and/or care services in programs currently financed by the Global Fund, illustrates how the Global Fund has started tooperationalize its 2012–2016 Strategy, focusing on high-impact interventions in those countries with the most acute need for ongoing external support.

Third, lives saved projections focused on the immediate, and full potential benefit of preventing deaths among patients treated. This perspective is relevant to the short- and medium-term Strategy for the Global Fund, which has an overall goal to save 10 million lives over 2012 to 2016 [7]. We estimated lives saved against a counterfactual of no TB treatment – an approach taken by the Global Fund in earlier estimations of lives saved through Global Fund-supported programs [33]. Recent WHO analyses instead focused on lives saved through the DOTS and Stop TB Strategies compared to TB control as it existed in 1995, i.e. under pre-DOTS standards, and accounting for the less than 100% cure rates typically observed in NTPs. This approach estimates around four-fold fewer deaths averted per treatment [10], [34]. Irrespective of the counterfactual chosen, the lives saved estimations are relevant to compare allocations among the three treatment categories and the four world regions within the horizon of the Global Fund 2012–2016 Strategy [7]. However, assessment of longer-term effects will require a broader perspective of the dynamics of both TB and HIV spread, including the impact of DOTS in preventing MDR-TB emergence, and of MDR-TB treatment in containing its subsequent spread [35], [36], [37], [38]. Such a dynamic projection could also consider the longer-term implications for financial and health system resource needs of starting TB patients on ART, where patients will have to be kept on ART after completing DOTS treatment.

Fourth, lacking reliable NTP finance data from certain countries, we assumed per-patient costs were fixed for all countries within a region, and − for both DOTS and MDR-TB treatment − the same across regions except EECA. In-depth costing studies undertaken in selected countries, however, suggest lower per-patient costs in India and China compared to Africa, and within sub-Saharan Africa higher cost in South Africa than in other high-TB burden countries [27], [29], [39], [40], [41], [42]. Within EECA, Russia with the highest unit cost for both DOTS and MDR-TB [9] and a large share in the region’s TB and MDR-TB burden increased the regionally weighted average unit costs (Table 1) by two-fold for DOTS and 13% for MDR-TB.

Fifth, we have not considered possible unintended consequences of moving to an allocation algorithm based on priority interventions (i.e. scenarios B and C). These will depend on co-investment by other donors and national governments, who may or may not shift contributions to non-Global Fund priority TB interventions also considered cost-effective according to WHO benchmarks [12], [13], [23]. More refined allocation algorithms that take into account each country’s actual NTP funding including domestic fiscal capacity to contribute to NTP needs were beyond the scope of the current analysis, and should be pursued in future. Ongoing improvements in national health financing reporting [20] will allow donors to further refine their allocation policies and maximize the return on investment. Regardless of allocation policies, not all health budget contributions will be used for the intended purpose. To reduce corrupt practices in its programs that have led to misappropriation of funds, the Global Fund is strengthening fiduciary oversight and financial control, including an expanded role in the process for civil society and affected communities [43].

### Conclusion

Despite these limitations, the projections illustrate how for given investments, the immediate mortality impact is determined by the balance in allocations between TB/HIV management mainly in Africa, with high return for low cost, and MDR-TB management mainly in EECA, with lowest return for highest cost. The future efficiency and effectiveness of TB treatment in high-cost region EECA will critically determine worldwide costs. In the short term, most lives will be saved through TB/HIV co-infection control in Africa. Investing in MDR-TB diagnosis and care, using the highly effective new technologies for diagnosis of TB and rifampicin-resistant TB, is needed to prevent MDR-TB, reduce substantial future costs of managing MDR-TB, and to reduce risk of unaffordability of TB control in longer-term. For the Global Fund, improved allocative efficiency of investments through proactive approaches that ‘inform demand’ would result in greater numbers of lives saved than might be with the prevailing funding model of responding to country demand. While an investment approach that fosters value for money improves allocative efficiency and aggregate health impact, two key considerations of the Global Fund’s 2012−2016 Strategy [7], it does not address equity and political dimensions of resource allocation, which must be considered. Alongside country financing gaps, absorptive and co-financing capacities and equity considerations, these allocation scenarios will help the Global Fund, other international donors and NTPs to implement more effective, evidence-based investment approaches focusing on highest-impact populations and interventions.

## Supporting Information

Table S1 **Grouping of countries into regions.** Abbreviations: I&C: India and China; EE & CA: EECA; Other L&MIC: other low and middle-income countries; SSA: sub-Saharan Africa.
(DOCX)

## References

[1] Stop TB Partnership, World Health Organization (2010). The Global Plan to Stop TB 2011–2015: Transforming the fight towards elimination of tuberculosis.. Geneva: Stop TB Partnership.

[2] The Global Fund to fight AIDS Tuberculosis, Malaria (2011). Global Fund-supported programs see strong results amid funding challenges [press release].. Geneva.

[3] World Health Organization (2011). Global tuberculosis control 2011..

[4] The Global Fund to fight AIDS Tuberculosis, Malaria (2012). Approved Grant Amounts and Disbursements: Disbursements in detail, Commitments and disbursements – summary..

[5] Kazatchkine MD (2010). Increased resources for the Global Fund, but pledges fall short of expected demand.. Lancet.

[6] The Global Fund to Fight AIDS Tuberculosis, Malaria (2011). The Global Fund Results report 2011: Making a difference.. Geneva.

[7] The Global Fund to fight AIDS Tuberculosis, Malaria (2011). The Global Fund Strategy 2012–2016: Investing for Impact.. Geneva.

[8] The Global Fund to Fight AIDS Tuberculosis, Malaria (2010). Replenishment 2011–2013. Resource scenarios 2011–2013. Funding the global fight against HIV/AIDS, tuberculosis and malaria.. Geneva.

[9] World Health Organization (2010). Global tuberculosis control 2010.. Geneva.

[10] Glaziou P, Floyd K, Korenromp EL, Sismanidis B, Bierrenbach A (2011). Lives saved by tuberculosis control and prospects for achieving the 2015 global target for reductions in tuberculosis mortality.. Bull WHO.

[11] Suarez PG, Floyd K, Portocarrero J, Alarcon E, Rapiti E (2002). Feasibility and cost-effectiveness of standardised second-line drug treatment for chronic tuberculosis patients: a national cohort study in Peru.. Lancet.

[12] Tupasi TE, Gupta R, Quelapio MI, Orillaza RB, Mira NR (2006). Feasibility and cost-effectiveness of treating multidrug-resistant tuberculosis: a cohort study in the Philippines.. PLoS Med.

[13] Fitzpatrick C, Floyd K (2012). A Systematic Review of the Cost and Cost Effectiveness of Treatment for Multidrug-Resistant Tuberculosis.. PharmacoEconomics 30: 63–80 10.2165/11595340-000000000-000000000.

[14] Straetemans M, Bierrenbach AL, Nagelkerke N, Glaziou P, van der Werf MJ (2010). The effect of tuberculosis on mortality in HIV positive people: a meta-analysis.. PLoS ONE.

[15] Tiemersma EW, van der Werf MJ, Borgdorff MW, Williams BG, Nagelkerke NJ (2011). Natural history of tuberculosis: duration and fatality of untreated pulmonary tuberculosis in HIV negative patients: a systematic review.. PLoS One.

[16] Mukadi JD, Maher D, Harries A (2001). Tuberculosis case fatality rates in high HIV prevalence populations in sub-Saharan Africa.. AIDS.

[17] Dye C, Scheele S, Dolin P, Pathania V, Raviglione MC (1999). Consensus statement. Global burden of tuberculosis: estimated incidence, prevalence, and mortality by country. WHO Global Surveillance and Monitoring Project.. Jama.

[18] Corbett EL, Watt CJ, Walker N, Maher D, Williams BG (2003). The growing burden of tuberculosis: global trends and interactions with the HIV epidemic.. Arch Intern Med.

[19] Franke MF, Appleton SC, Bayona J, Arteaga F, Palacios E (2008). Risk factors and mortality associated with default from multidrug-resistant tuberculosis treatment.. Clin Infect Dis.

[20] Floyd K, Pantoja A, Dye C (2007). Financing tuberculosis control: the role of a global financial monitoring system.. Bull World Health Organ.

[21] World Health Organization, Stop TB Partnership (2011). The Global Plan to Stop TB 2011–2015..

[22] Jia ZW, Cheng SM, Li ZJ, Du X, Huang F (2010). Combining domestic and foreign investment to expand tuberculosis control in China.. PLoS Med.

[23] Baltussen R, Floyd K, Dye C (2005). Cost effectiveness analysis of strategies for tuberculosis control in developing countries.. BMJ.

[24] Lawn SD, Churchyard G (2009). Epidemiology of HIV-associated tuberculosis.. Curr Opin HIV AIDS.

[25] International Monetary Fund (2010). World Economic Outlook Database: Gross domestic product per capita, current prices (U.S. dollars)..

[26] Atun RA, Samyshkin Y, Drobniewski F, Balabanova Y, Fedorin I (2006). Costs and outcomes of tuberculosis control in the Russian Federation: retrospective cohort analysis.. Health Policy Plan.

[27] Floyd K, Hutubessy R, Samyshkin Y, Korobitsyn A, Fedorin I (2006). Health-systems efficiency in the Russian Federation: tuberculosis control.. Bull World Health Organ.

[28] Balabanova Y, Drobniewski F, Fedorin I, Zakharova S, Nikolayevskyy V (2006). The Directly Observed Therapy Short-Course (DOTS) strategy in Samara Oblast, Russian Federation.. Respir Res.

[29] Floyd K, Arora VK, Murthy KJ, Lonnroth K, Singla N (2006). Cost and cost-effectiveness of PPM-DOTS for tuberculosis control: evidence from India.. Bull World Health Organ.

[30] Lal SS, Uplekar M, Katz I, Lonnroth K, Komatsu R (2011). Global Fund financing of public-private mix approaches for delivery of tuberculosis care.. Trop Med Int Health.

[31] The Global Fund to Fight AIDS Tuberculosis, Malaria (2011). Policy on eligibility criteria, counterpart financing requirements, and prioritization of proposals for funding from the Global Fund. Geneva.. GF/B23/14, Attachment 1 GF/B23/14, Attachment 1.

[32] The Global Fund to fight AIDS Tuberculosis, Malaria (2012). Transitional Funding Mechanism..

[33] Komatsu R, Korenromp EL, Low-Beer D, Watt C, Dye C (2010). Lives saved by Global Fund-supported HIV/AIDS, tuberculosis and malaria programs: estimation approach and results between 2003 and end-2007.. BMC Inf Dis.

[34] Korenromp EL, Bierrenbach AL, Williams BG, Dye C (2009). The measurement and estimation of tuberculosis mortality.. Int J Tuberc Lung Dis.

[35] Dye C, Williams BG (2009). Slow elimination of multidrug-resistant tuberculosis.. Sci Transl Med.

[36] Dye C, Williams BG (2010). The population dynamics and control of tuberculosis.. Science.

[37] Williams BG, Granich R, De Cock KM, Glaziou P, Sharma A (2010). Antiretroviral therapy for tuberculosis control in nine African countries.. Proc Natl Acad Sci U S A.

[38] Cohen T, Dye C, Colijn C, Williams B, Murray M (2009). Mathematical models of the epidemiology and control of drug-resistant TB.. Expert Rev Respir Med.

[39] Nganda B, Wang’ombe J, Floyd K, Kangangi J (2003). Cost and cost-effectiveness of increased community and primary care facility involvement in tuberculosis care in Machakos District, Kenya.. Int J Tuberc Lung Dis.

[40] Okello D, Floyd K, Adatu F, Odeke R, Gargioni G (2003). Cost and cost-effectiveness of community-based care for tuberculosis patients in rural Uganda.. Int J Tuberc Lung Dis.

[41] Sinanovic E, Floyd K, Dudley L, Azevedo V, Grant R (2003). Cost and cost-effectiveness of community-based care for tuberculosis in Cape Town, South Africa.. Int J Tuberc Lung Dis.

[42] Floyd K, Pantoja A (2008). Financial resources required for tuberculosis control to achieve global targets set for 2015.. Bull World Health Organ.

[43] The Global Fund to fight AIDS Tuberculosis, Malaria (2011). Report of the Comprehensive Reform Working Group. Geneva.. GF/B23/13 GF/B23/13.

[44] World Health Organization (2006, with 2011 update.) TB Planning and Budgeting tool.. Geneva.

[45] Currie CS, Floyd K, Williams BG, Dye C (2005). Cost, affordability and cost-effectiveness of strategies to control tuberculosis in countries with high HIV prevalence.. BMC Public Health.

[46] World Health Organization (2010). Antiretroviral therapy for HIV infection in adults and adolescents – Recommendations for a public health approach (2010 version).. Geneva.

[47] Stover J, Bollinger L, Avila C (2011). Estimating the impact and cost of the WHO 2010 recommendations for antiretroviral therapy.. AIDS Research and Treatment Article ID 738271.

[48] Pitter C, Kahn JG, Marseille E, Lule JR, McFarland DA (2007). Cost-effectiveness of cotrimoxazole prophylaxis among persons with HIV in Uganda.. J Acquir Immune Defic Syndr.

[49] Zumla A, Atun R, Maeurer M, Mwaba P, Ma Z (2011). Viewpoint: Scientific dogmas, paradoxes and mysteries of latent Mycobacterium tuberculosis infection.. Trop Med Int Health.

