# Supplementary material for: Implementing the Global Plan to Stop TB, 2011–2015 – Optimizing Allocations and the Global Fund’s Contribution: A Scenario Projections Study
Source: PLoS One. 2012 Jun 18;7(6):e38816. doi: 10.1371/journal.pone.0038816 (PMC3377722; doi:10.1371/journal.pone.0038816)
Supplement: Table S1 — Grouping of countries into regions. Abbreviations: I&C: India and China; EE & CA: EECA; Other L&MIC: other low and middle-income countries; SSA: sub-Saharan Africa. (DOCX) [file pone.0038816.s001.docx]

# Supporting Information: table S1.

| *Region* | *Country* | *IS03* |
| --- | --- | --- |
| I&C | China | CHN |
| I&C | India | IND |
| EE & CA | Armenia | ARM |
| EE & CA | Azerbaijan | AZE |
| EE & CA | Belarus | BLR |
| EE & CA | Bulgaria | BGR |
| EE & CA | Georgia | GEO |
| EE & CA | Kazakhstan | KAZ |
| EE & CA | Kyrgyzstan | KGZ |
| EE & CA | Latvia | LVA |
| EE & CA | Lithuania | LTU |
| EE & CA | Republic of Moldova | MDA |
| EE & CA | Romania | ROU |
| EE & CA | Russian Federation | RUS |
| EE & CA | Tajikistan | TJK |
| EE & CA | Turkmenistan | TKM |
| EE & CA | Ukraine | UKR |
| EE & CA | Uzbekistan | UZB |
| Other L&MIC | Afghanistan | AFG |
| Other L&MIC | Albania | ALB |
| Other L&MIC | Algeria | DZA |
| Other L&MIC | American Samoa | ASM |
| Other L&MIC | Argentina | ARG |
| Other L&MIC | Bangladesh | BGD |
| Other L&MIC | Belize | BLZ |
| Other L&MIC | Bhutan | BTN |
| Other L&MIC | Bolivia (Plurinational State of) | BOL |
| Other L&MIC | Bosnia and Herzegovina | BIH |
| Other L&MIC | Brazil | BRA |
| Other L&MIC | Cambodia | KHM |
| Other L&MIC | Chile | CHL |
| Other L&MIC | Colombia | COL |
| Other L&MIC | Comoros | COM |
| Other L&MIC | Cook Islands | COK |
| Other L&MIC | Costa Rica | CRI |
| Other L&MIC | Cuba | CUB |
| Other L&MIC | Democratic People's Republic of Korea | PRK |
| Other L&MIC | Dominica | DMA |
| Other L&MIC | Dominican Republic | DOM |
| Other L&MIC | Ecuador | ECU |
| Other L&MIC | Egypt | EGY |
| Other L&MIC | El Salvador | SLV |
| Other L&MIC | Estonia | EST |
| Other L&MIC | Fiji | FJI |
| Other L&MIC | Grenada | GRD |
| Other L&MIC | Guatemala | GTM |
| Other L&MIC | Guyana | GUY |
| Other L&MIC | Haiti | HTI |
| Other L&MIC | Honduras | HND |
| Other L&MIC | Indonesia | IDN |
| Other L&MIC | Iran (Islamic Republic of) | IRN |
| Other L&MIC | Iraq | IRQ |
| Other L&MIC | Jamaica | JAM |
| Other L&MIC | Jordan | JOR |
| Other L&MIC | Kiribati | KIR |
| Other L&MIC | Lao Peoples Dem. Rep. | LAO |
| Other L&MIC | Lebanon | LBN |
| Other L&MIC | Libyan Arab Jamahiriya | LBY |
| Other L&MIC | Malaysia | MYS |
| Other L&MIC | Maldives | MDV |
| Other L&MIC | Marshall Islands | MHL |
| Other L&MIC | Mexico | MEX |
| Other L&MIC | Micronesia (Fed. States of) | FSM |
| Other L&MIC | Mongolia | MNG |
| Other L&MIC | Montenegro | MNE |
| Other L&MIC | Morocco | MAR |
| Other L&MIC | Myanmar | MMR |
| Other L&MIC | Nauru | NRU |
| Other L&MIC | Nepal | NPL |
| Other L&MIC | Nicaragua | NIC |
| Other L&MIC | Niue | NIU |
| Other L&MIC | Pakistan | PAK |
| Other L&MIC | Palau | PLW |
| Other L&MIC | Panama | PAN |
| Other L&MIC | Papua New Guinea | PNG |
| Other L&MIC | Paraguay | PRY |
| Other L&MIC | Peru | PER |
| Other L&MIC | Philippines | PHL |
| Other L&MIC | Poland | POL |
| Other L&MIC | Saint Kitts and Nevis | KNA |
| Other L&MIC | Saint Lucia | LCA |
| Other L&MIC | Saint Vincent and the Grenadines | VCT |
| Other L&MIC | Samoa | WSM |
| Other L&MIC | Serbia | SRB |
| Other L&MIC | Solomon Islands | SLB |
| Other L&MIC | Sri Lanka | LKA |
| Other L&MIC | Suriname | SUR |
| Other L&MIC | Syrian Arab Republic | SYR |
| Other L&MIC | Thailand | THA |
| Other L&MIC | Former Yug. Rep. Macedonia | MKD |
| Other L&MIC | Timor-Leste | TLS |
| Other L&MIC | Tokelau | TKL |
| Other L&MIC | Tonga | TON |
| Other L&MIC | Tunisia | TUN |
| Other L&MIC | Turkey | TUR |
| Other L&MIC | Tuvalu | TUV |
| Other L&MIC | Uruguay | URY |
| Other L&MIC | Vanuatu | VUT |
| Other L&MIC | Venezuela (Bolivarian Re. of) | VEN |
| Other L&MIC | Viet Nam | VNM |
| Other L&MIC | Wallis and Futuna Islands | WLF |
| Other L&MIC | West Bank and Gaza Strip | PSE |
| Other L&MIC | Yemen | YEM |
| SSA | Angola | AGO |
| SSA | Benin | BEN |
| SSA | Botswana | BWA |
| SSA | Burkina Faso | BFA |
| SSA | Burundi | BDI |
| SSA | Cameroon | CMR |
| SSA | Cape Verde | CPV |
| SSA | Central African Republic | CAF |
| SSA | Chad | TCD |
| SSA | Congo | COG |
| SSA | Côte d'Ivoire | CIV |
| SSA | Democratic Republic of the Congo | COD |
| SSA | Djibouti | DJI |
| SSA | Eritrea | ERI |
| SSA | Ethiopia | ETH |
| SSA | Gabon | GAB |
| SSA | Gambia | GMB |
| SSA | Ghana | GHA |
| SSA | Guinea | GIN |
| SSA | Guinea-Bissau | GNB |
| SSA | Kenya | KEN |
| SSA | Lesotho | LSO |
| SSA | Liberia | LBR |
| SSA | Madagascar | MDG |
| SSA | Malawi | MWI |
| SSA | Mali | MLI |
| SSA | Mauritania | MRT |
| SSA | Mauritius | MUS |
| SSA | Mozambique | MOZ |
| SSA | Namibia | NAM |
| SSA | Niger | NER |
| SSA | Nigeria | NGA |
| SSA | Rwanda | RWA |
| SSA | Sao Tome and Principe | STP |
| SSA | Senegal | SEN |
| SSA | Seychelles | SYC |
| SSA | Sierra Leone | SLE |
| SSA | Somalia | SOM |
| SSA | South Africa | ZAF |
| SSA | Sudan | SDN |
| SSA | Swaziland | SWZ |
| SSA | Togo | TGO |
| SSA | Uganda | UGA |
| SSA | United Republic of Tanzania | TZA |
| SSA | Zambia | ZMB |
| SSA | Zimbabwe | ZWE |
